# Upregulation of T-type Ca^2+^ channels in long-term diabetes determines increased excitability of a specific type of capsaicin-insensitive DRG neurons

**DOI:** 10.1186/s12990-015-0028-z

**Published:** 2015-05-20

**Authors:** Dmytro E Duzhyy, Viacheslav Y Viatchenko-Karpinski, Eugen V Khomula, Nana V Voitenko, Pavel V Belan

**Affiliations:** Department of General Physiology of the CNS and State Key Laboratory of Molecular and Cellular Biology, Bogomoletz Institute of Physiology of National Academy of Science of Ukraine, 4 Bogomoletz street, 01024 Kyiv, Ukraine; International Center of Molecular Physiology of National Academy of Science of Ukraine, 4 Bogomoletz street, 01024 Kyiv, Ukraine

**Keywords:** Diabetes, Pain, T-type calcium channels, DRG neurons, Excitability, Capsaicin-insensitive

## Abstract

**Background:**

Previous studies have shown that increased excitability of capsaicin-sensitive DRG neurons and thermal hyperalgesia in rats with short-term (2–4 weeks) streptozotocin-induced diabetes is mediated by upregulation of T-type Ca^2+^ current. In longer–term diabetes (after the 8th week) thermal hyperalgesia is changed to hypoalgesia that is accompanied by downregulation of T-type current in capsaicin-sensitive small-sized nociceptors. At the same time pain symptoms of diabetic neuropathy other than thermal persist in STZ-diabetic animals and patients during progression of diabetes into later stages suggesting that other types of DRG neurons may be sensitized and contribute to pain. In this study, we examined functional expression of T-type Ca^2+^ channels in capsaicin-insensitive DRG neurons and excitability of these neurons in longer-term diabetic rats and in thermally hypoalgesic diabetic rats.

**Results:**

Here we have demonstrated that in STZ-diabetes T-type current was upregulated in capsaicin-insensitive low-pH-sensitive small-sized nociceptive DRG neurons of longer-term diabetic rats and thermally hypoalgesic diabetic rats. This upregulation was not accompanied by significant changes in biophysical properties of T-type channels suggesting that a density of functionally active channels was increased. Sensitivity of T-type current to amiloride (1 mM) and low concentration of Ni^2+^ (50 μM) implicates prevalence of Ca_v_3.2 subtype of T-type channels in the capsaicin-insensitive low-pH-sensitive neurons of both naïve and diabetic rats. The upregulation of T-type channels resulted in the increased neuronal excitability of these nociceptive neurons revealed by a lower threshold for action potential initiation, prominent afterdepolarizing potentials and burst firing. Sodium current was not significantly changed in these neurons during long-term diabetes and could not contribute to the diabetes-induced increase of neuronal excitability.

**Conclusions:**

Capsaicin-insensitive low-pH-sensitive type of DRG neurons shows diabetes-induced upregulation of Ca_v_3.2 subtype of T-type channels. This upregulation results in the increased excitability of these neurons and may contribute to nonthermal nociception at a later-stage diabetes.

## Background

Hyperalgesia and allodynia to mechanical and thermal stimuli are frequent pain symptoms at an early stage of peripheral diabetic neuropathy, which involves about 66 % of diabetic patients [1, 2]. These symptoms are caused by sensitization of sensory transduction pathways that involves modulation of ligand- and voltage-gated ion channels [3]. At early stages of STZ-induced diabetes upregulation of T-type channels in small- and medium-sized nociceptive DRG neurons was found to increase their excitability contributing to thermal and mechanical hyperalgesia [4, 5]. In longer-term diabetes thermal hyperalgesia is changed to hypoalgesia in case of animal models of diabetes and in diabetic patients [6, 7] (for review see [8]). It is accompanied by downregulation of TRPV1 [9] and T-type channels in the capsaicin-sensitive (caps^+^) subpopulation of small-sized nociceptors [10, 11] most likely leading to decreased excitability of these neurons. At the same time manifestations of pain of different origin, e.g., spontaneous pain, mechanical hyperalgesia, and tactile allodynia may persist [8, 12] suggesting sensitization of capsaicin-insensitive (caps^−^) mechanical and/or chemical nociceptors. In particular, inflammation and acidosis developing under diabetic conditions [13–15] may cause sensitization of capsaicin-insensitive low pH-sensitive (caps^−^lpH^+^) DRG neurons through the pathway involving ASICs [16, 17]. Modulation of Ca^2+^ and Na^+^ voltage-gated ion channels leading to increased excitability of nociceptive neurons is a frequent hallmark of sensitization in diabetes and painful neuropathies [4, 9, 10, 18, 19]. Thus, the objective of this study was to find out whether nociceptive caps^−^lpH^+^ DRG neurons show upregulation of Ca^2+^ and Na^+^ voltage-gated channels and increased excitability in longer-term diabetes when thermal sensory modality is downregulated. Here, we report on a specific caps^−^lpH^+^ type of DRG neurons that exhibits increased excitability in the longer-term STZ-diabetic rats and is likely involved in nonthermal nociception at the later-stage diabetes.

## Results

### Isolation of capsaicin-insensitive low-pH-sensitive nociceptive DRG neurons

**Fig. 1:**
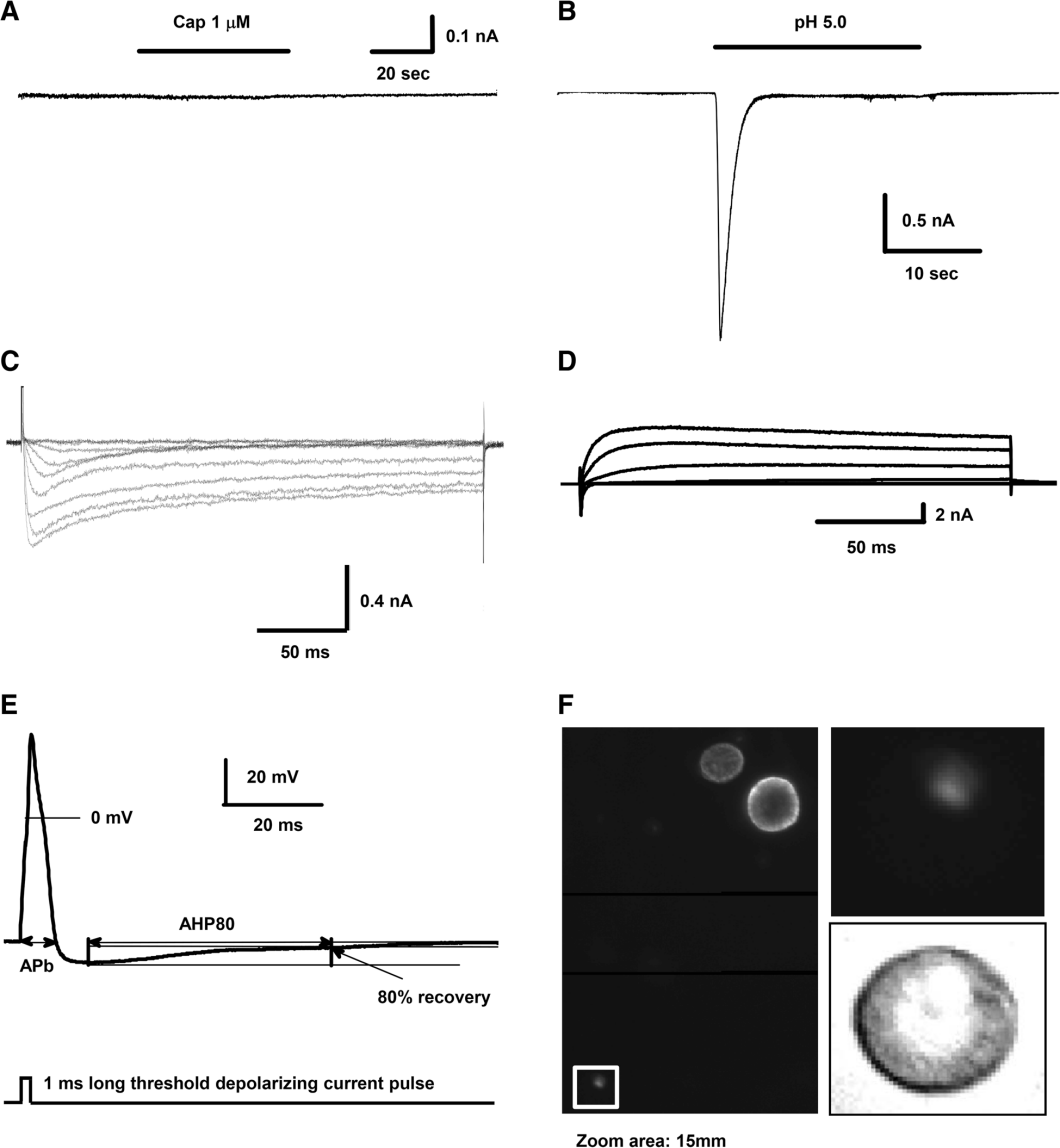
Electrophysiological and pharmacological properties of the caps^−^lpH^+^ small-sized DRG neurons studied for diabetes-induced changes in T-type current. **a** A representative trace showing absence of capsaicin-induced current in the caps^−^lpH^+^ neurons. **b** A representative trace demonstrating substantial low-pH-induced current in the caps^−^lpH^+^ neurons. **c** Representative traces of Ba^2+^ currents demonstrating expression of T-type channels in the caps^−^lpH^+^ neurons. The currents were evoked by voltage depolarizing steps from a holding potential of −100 mV to −80 through −10 mV in 10 mV increments. **d** Representative traces of total currents recorded in the caps^−^lpH^+^ neurons that included Na^+^, Ca^2+^ and K^+^ components. The currents were evoked by voltage steps from a holding potential of −100 to −60 through 40 mV in 20 mV increments. **e** At the top, a representative trace of AP evoked by a threshold 1 millisecond-long current pulse shown at the bottom. APb is an action potential duration at the base. AHP80 is the time required for the AHP to decay to 80 % of its peak value. **f** An example demonstrating that the caps^−^lpH^+^ neurons are IB4-negative. A fluorescent image of DRG neurons stained for IB4 is shown in the left rectangle. Two IB4-positive small DRG neurons are clearly visible in the top right corner of the image. White box in a bottom left corner of the image indicates an area where IB4-negative caps^−^lpH^+^ neuron is located. Fluorescent and transmitted light images of the boxed caps^−^lpH^+^ neuron are presented in the top and bottom right squares, respectively. The IB4-negative caps^−^lpH^+^ neuron is outlined with a white dashed circle on fluorescent images and a black dashed circle on transmitted light image. No IB4 fluorescence is visible on the plasma membrane of this caps^−^lpH^+^ neuron. A scale bar is 15 μm. This neuron had a K^+^ “current signature” specific for the caps^−^lpH^+^ DRG neurons

The scope of search for the caps^−^lpH^+^ neurons was limited to a population of small-sized DRG neurons (<25 pF) that provided enrichment in C-fibre nociceptors [20], and to L4-L6 ganglia where neurons innervating hind paws are localized. Isolation of the caps^−^lpH^+^ nociceptive DRG neurons was based on classification of DRG neuronal types [21]. First, separate types of neurons were identified in the aforementioned subpopulation of DRG neurons based on size and set of K^+^ current characteristics [21] (activation threshold, presence of A-type fast-inactivating component, K^+^ current inactivation time constant (τ_inh_)). Next, these types were tested for expression of capsaicin-activated and transient low-pH-activated currents, and for fast-inactivating T-type current involved in abnormalities of thermal and mechanical sensitivity under diabetic conditions [4, 5]. The caps^−^lpH^+^ DRG neurons (Fig 1a, b) expressing fast-inactivating T-type current (Fig 1c) selected in this way have appeared to be the members of one type with a distinct set of physical and electrical parameters. Neurons of this type had a very small size (their capacitance was in a range 10–21 pF with a mean value of 15.4 ± 0.7 pF, *n* = 19, four rats). They also expressed K^+^ current with a specific “current signature”: (i) an activation threshold of −20 mV, (ii) no obvious A-type component, and (iii) slow inactivation (τ_inact_ fell within a range of 53–419 ms with a mean value of 209 ± 31 ms, *n* = 19, four rats; Fig 1d).

In order to verify that the neuronal size, presence of fast-inactivating T-type current (Fig 1c) and K^+^ current characteristics mentioned above (Fig 1d) represent sufficient criterion for identifying the caps^−^lpH^+^ DRG neurons, cells (*n* = 8, three rats) having these characteristics were selected among small-sized DRG neurons and challenged by applications of capsaicin and low pH. All of them were found to be capsaicin-insensitive (TRPV1 lacking) and low-pH sensitive implying that size and specific current profile were sufficient to identify the caps^−^lpH^+^ DRG neurons. In the following electrophysiological experiments the caps^−^lpH^+^ nociceptive DRG neurons were routinely selected based on their size, presence of fast-inactivating T-type current and K^+^ current characteristics (Fig 1c, d). In some experiments the caps^−^lpH^+^ neurons were additionally challenged with capsaicin in order to check their TRPV1 lacking phenotype.

The transient low-pH-induced current in the caps^−^lpH^+^ neurons (Fig 1b) was sensitive to amiloride (data not shown), which is a hallmark of ASIC channels [22, 23]. Characteristic rise time of low-pH-activated current (time from a first deflection to 90 % of peak amplitude) at pH jump from 7.4 to 5.0 was 522 ± 67 ms (*n* = 8, three rats) and its inactivation time constant was 847 ± 72 ms (*n* = 8, three rats). This relatively slow inactivation of low-pH-induced current in the caps^−^lpH^+^ neurons suggests that it might be potentially mediated by Ca^2+^ permeable ASIC1a homomultimers [24].

Next, we tested whether the caps^−^lpH^+^ neurons were nociceptive. It has been previously shown that AP overshoot, AP duration at the base (APb), and afterhyperpolarization (AHP) duration at 80 % recovery (AHP80) have the largest values for nociceptive neurons among the whole population of DRG neurons [25]. AP overshoot, APb and AHP80 of APs induced in the caps^−^lpH^+^ neurons in response to a short (1 ms) threshold current stimulation were 42 ± 3 mV, 5.8 ± 0.5 ms and 69 ± 4 ms, correspondently (*n* = 7, three rats; Fig 1e). These values were close (for AP duration) or substantially larger (for AP overshoot, AHP80) than respective values for C-fibre nociceptive neurons (AP overshoot ~ 22 mV, APb ~ 6 ms and AHP80 ~ 22 ms [25]) confirming that the caps^−^lpH^+^ neurons are likely nociceptive.

For additional characterization the caps^−^lpH^+^ neurons were checked for binding of isolectin B4 (IB4) widely used to discriminate GDNF-dependent nonpeptidergic (IB4 positive) from NGF-dependent peptidergic (IB4 negative) DRG neurons [26, 27], and appeared to be IB4 negative (Fig 1 f, 7 out of 7 neurons tested, three rats). Thus, our results allow to classify the caps^−^lpH^+^ neurons studied in this work as peptidergic C-fiber nociceptors lacking TRPV1 receptors and expressing ASIC channels.

### Increased functional expression of T-type channels in the caps^−^lpH^+^ neurons of longer-term diabetic rats

**Fig. 2:**
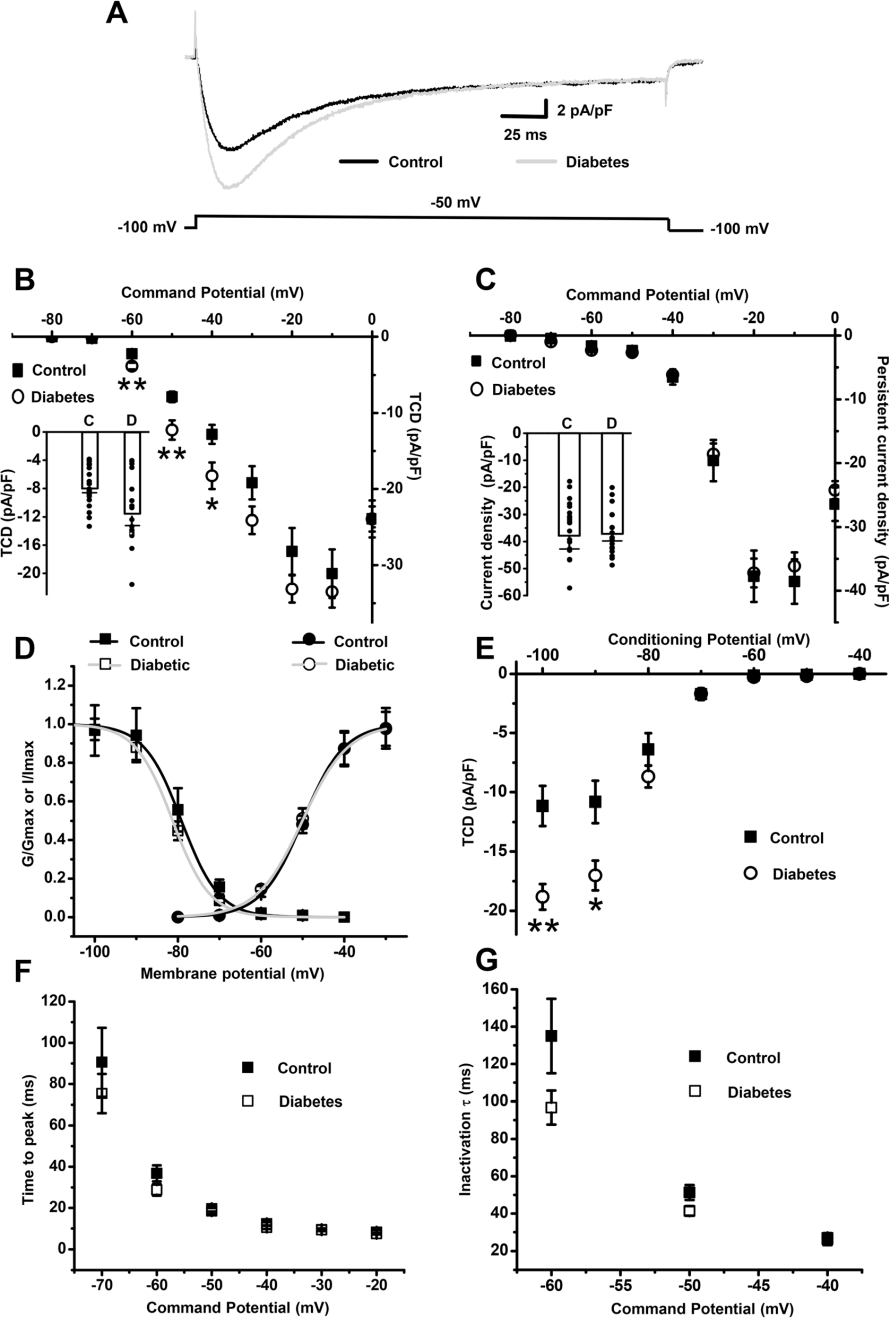
T-type channels are specifically upregulated in the caps^−^lpH^+^ neurons of longer-term diabetic rats without changes in their biophysical properties. **a** Representative traces of Ba^2+^ current recorded in the caps^−^lpH^+^ neurons of control and diabetic rats. Currents were evoked by a depolarization step from a holding potential of −100 to −50 mV. **b** Diabetes-induced upregulation of T-type channels is revealed at the plot of the transient current density, TCD, versus a depolarizing voltage step in the activation protocol. An insert demonstrates TCD amplitude for each tested neuron and their mean values (boxes) with standard errors (upper whiskers) in the control (c) and diabetic (d) neurons at a depolarization step to −50 mV. **c** HVA currents were not changed under diabetic conditions as revealed at the plot of persistent Ba^2+^ current versus depolarizing step in the activation protocol. The averaged value of Ba^2+^ current during last 10 ms of depolarization step was taken as a persistent current amplitude. Current densities are not significantly different between diabetes and control in a range of voltage steps from −30 to 0 mV where HVA currents are main contributors to the persistent current (p > 0.05). An insert demonstrates persistent current density amplitude for each tested neuron and their mean values (boxes) with standard errors (upper whiskers) in the control (c) and diabetic (d) neurons at a depolarization step to −20 mV. **d** Biophysical properties of T-type channels of the caps^−^lpH^+^ neurons are not changed in longer-term diabetes. Inactivation of normalized transient current (left curves) calculated for experimental results depicted in E; activation of normalized transient current conductance (right curves) for the experimental results depicted in B. **e** Diabetes-induced upregulation of T-type channels is revealed in a voltage-dependent inactivation protocol for evoking transient Ba^2+^ currents consisted of depolarization steps to a test potential of −40 mV (250 ms) from a holding potential ranging from −100 to −40 mV (3.5 s) with 10 mV increments. **f** Time to peak, TTP, for Ba^2+^ currents recorded in a protocol used to estimate the transient current activation. **g** Inactivation time constant, τ, obtained from a single-exponential fit of the decaying phase of the currents evoked in the activation protocol. Numbers of cells: 19 cells from 4 rats in control group and 17 cells from 4 rats in diabetic group. Data are expressed as Mean ± S.E.M. *p < 0.02, **p < 0.001

The caps^−^lpH^+^ DRG neurons are likely to be peptidergic C-fiber nociceptors lacking TRPV1 receptors and expressing both ASIC channels and fast-inactivating T-type channels. It suggests their possible involvement in pain symptoms (other than thermal hyperalgesia) of longer-term (12 weeks) diabetic neuropathy [7, 8, 12]. Since T-type current upregulation in small nociceptive DRG neurons is involved in diabetes-induced mechanical hyperalgesia [4], we checked whether such upregulation occurs in the caps^−^lpH^+^ DRG neurons of longer-term (9–13 weeks) diabetic rats, for which multiple pain symptoms including mechanical hyperalgesia have been demonstrated [7, 8, 12]. Blood glucose level and body weight of diabetic animals at this stage of diabetes were significantly different from the control group (Table 1). Mean capacitance of these neurons (16.6 ± 0.4 pF, *n* = 17, four rats) was not significantly different compared to control (15.4 ± 0.7 pF, *n* = 19, four rats; *P* > 0.12) indicating that the caps^−^lpH^+^ neurons preserved their size in longer-term diabetes. Changes in Ca^2+^ current density were tracked separately for transient and persistent components (Fig 2a-c, see section Analysis in Methods for the definition). In a range of voltage steps from −100 mV to −80 through −40 mV both components are predominantly represented by T-type current. Starting from a voltage step to −30 mV N-type current (having the inactivation kinetics intermediate between T-type and L-type current) substantially contributes to the transient and persistent components of Ba^2+^ current [28]. L-type current (having the slowest inactivation kinetics [28]) did also contribute to the persistent component at voltage steps from −30 to 0 mV (data not shown).

**Table 1:** Blood glucose level and body weight of experimental rats

Group	Old rats, T-current measurements^a^		Old rats, Na-current measurements^b^		2-3 months old rats^c^		
	Control	Diabetic 9-13 weeks	Control	Diabetic 9-13 weeks	Control	Diabetic 6-7 weeks	
						Hypoalgesic	Hyperalgesic
Body weight, g	325 + 25	212 + 20^d^	323 + 12	213 + 14^d^	153 + 4	128 + 11^e^	125 + 8^e^
Blood glucose level, mM	4.5 + 0.3	27 + 2^f^	4.4 + 0.2	28 + 2^f^	4.3 + 0.3	27 + 2^g^	26 + 2^g^
n	4	4	3	3	3	3	3

^a^Animals (8 months old) were used to measure T-type current and excitability parameters

^b^Animals (8 months old) were used to measure Na^+^ current

^c^Animals were used to measure T-type current

^d^ANOVA one way test, *p* < 0.01

^e^There was no significant difference between the group of diabetic hypoalgesic or hyperalgesic and control rats (*p* > 0.08, ANOVA one way test)

^f^Student’s t-test: *p* < 0.001 for each control and corresponding diabetic group

^g^Student’s t-test with α level adjusted with Bonferroni’s procedure: *p* < 0.005 when diabetic hypoalgesic and hyperalgesic groups were compared with the same control group

It turned out that there was a significant increase of transient current density (TCD) in the caps^−^lpH^+^ neurons in diabetes compared to control at depolarization steps from −100 mV to −60 through −40 mV (Fig 2a,b). For example, TCD at a test step to −60 mV, at which T-type current solely contributed to Ca^2+^ current, was 1.7 times higher in diabetic neurons compared to control. At the same time the persistent current density did not differ significantly between neurons of diabetic and control rats in a range of voltage steps from −30 to 0 mV (Fig 2c) indicating that HVA currents were not changed under diabetic conditions.

Fitting of steady-state activation of T-type current was further used to evaluate T-type channel remodeling in the caps^−^lpH^+^ neurons of diabetic animals (see section Analysis in the Materials for details). This fitting demonstrated that the maximal conductance of T-type current, *G*_*max*_, was significantly increased in the caps^−^lpH^+^ neurons isolated from diabetic rats (0.34 ± 0.03 pA/(pF*mV) versus 0.22 ± 0.03 pA/(pF*mV) in control; *P* < 0.005), while other fitting parameters, *V*_*50*_ and *k*, were not significantly changed (*V*_*50*_ = −50 ± 0.5 mV, *k* = 5.5 ± 0.3 mV compared to *V*_*50*_ = −50 ± 0.4 mV, *k* = 5.0 ± 0.2 mV for control; *P* > 0.95 and *P* > 0.13 for *V*_*50*_ and *k*, correspondently, Fig 2d). TCDs in a steady-state inactivation protocol were also significantly different between neurons from diabetic and control rats at low conditional potentials (−100 mV and −90 mV; Fig 2e) (see section Analysis in the Materials and Methods for details). Fitting data with a function *I*_*max*_/(1 + exp((*V*-*V*_*50*_)/*k*)) gave a significantly higher value for *I*_*max*_ in diabetes (−19.4 ± 1.0 pA/pF) compared to control (−12.4 ± 1.7 pA/pF; *P* < 0.005). Other fitted parameters did not significantly differ between control and diabetic conditions (control, *V*_*50*_ = −79.1 ± 0.9 mV, *k* = 4.5 ± 0.2 mV; diabetes, *V*_*50*_ = −81.3 ± 0.9 mV, *k* = 4.5 ± 0.2; *P* > 0.2 and *P* > 0.9 for *V*_*50*_ and *k*, correspondently, Fig 2d).

Kinetic parameters of Ba^2+^ current measured in a steady-state activation protocol, time to peak (TTP) and inactivation time constant (τ), were not significantly changed in diabetes compared to control (TTP from 19.6 ± 1.4 to 18.5 ± 1.5 ms, *P* > 0.5, and τ from 51.3 ± 4.0 to 41.4 ± 2.6 ms, *P* > 0.05, at a depolarization step to −50 mV, Fig 2 f, g).

Thus, functional expression of T-type Ca^2+^ channels, which is characterized by *G*_*max*_ and *I*_*max*_ was increased in the caps^−^lpH^+^ DRG neurons of longer-term diabetic rats. At the same time the gating parameters of T-type channels (*V*_*50*_ and *k* for activation, TTP and τ) were not changed under diabetic conditions.

### Upregulation of T-type current in the caps^−^lpH^+^ neurons of 6-7-weeks STZ-diabetic rats with thermal hypoalgesia

Our current data (see previous section) have demonstrated upregulation of T-type current in the caps^−^lpH^+^ neurons in the longer-term (9–13 weeks) diabetic animals. Previous results have shown that at an earlier stage of diabetes (6–7 weeks) T-type current is downregulated in caps^+^ DRG neurons of thermally hypoalgesic animals [10]. It remained unclear whether differential expression of T-type current in the caps^+^ [10] and caps^−^lpH^+^ (previous section) neurons was due to different sensory modalities of these neuronal types, or due to differences in the age of animals and/or duration of diabetes. To exclude the latter reasons we measured T-type current in the caps^−^lpH^+^ DRG neurons of thermally hypoalgesic rats of the same age (9–10 weeks old) and at the same stage of diabetes (6–7 weeks), for which downregulation of T-type current in the caps^+^ DRG neurons has been established. Besides, upregulation of T-type current in the caps^−^lpH^+^ neurons of thermally hypoalgesic rats would also suggest that hyperexcitability of these neurons may underlie diabetic pain symptoms other than thermal (spontaneous pain, mechanical hyperalgesia, and tactile allodynia), that are observed at a later (6–13 weeks) stage of diabetes development [7, 8, 12].

**Fig. 3:**
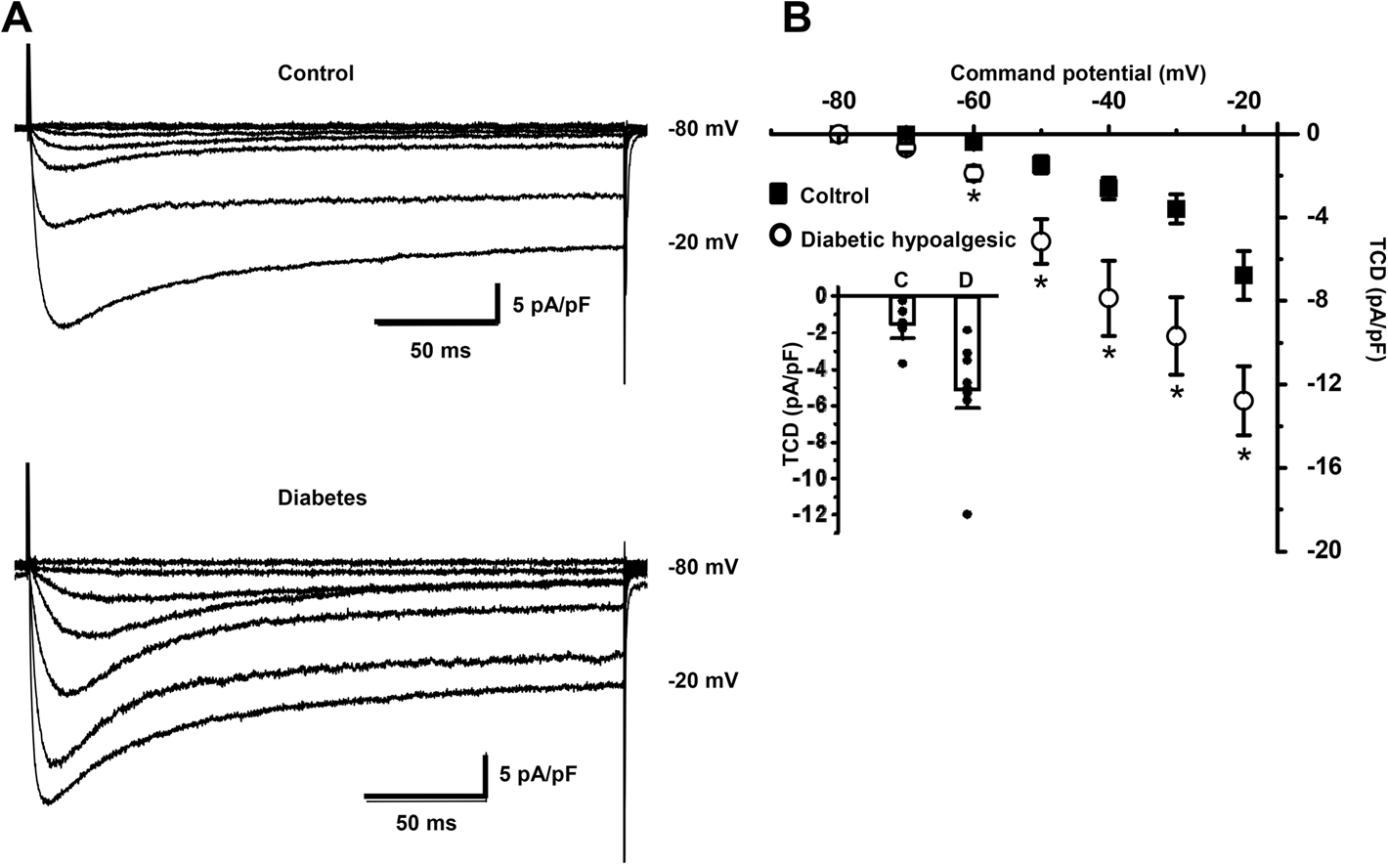
Diabetes-induced upregulation of T-type channels in the caps^−^lpH^+^ neurons of hypoalgesic subgroup of diabetic rats. **a** Representative traces of Ba^2+^ current in the caps^−^lpH^+^ neurons isolated from the control (upper traces), hypoalgesic (lower traces) diabetic rats. **b** Significant increase of TCD was observed in the caps^−^lpH^+^ neurons of diabetic compared to control rats in a range of −60 - -0 mV where Ba^2+^ current is mediated by T-type channels (*n* = 8 and *n* = 7, from three animals each, for control and diabetic groups, respectively; *p < 0.05). The caps^−^lpH^+^ neurons were isolated from diabetic animals after 6 weeks of STZ-induced diabetes. The currents were evoked by depolarizing steps from a holding potential of −100 mV to −80 through 0 mV in 10 mV increments. An insert demonstrates TCD amplitude for each tested neuron and their mean values (boxes C - for the control and D - for the diabetes) with standard errors (upper whiskers) at a depolarization step to −50 mV

Subgroups of hypoalgesic and hyperalgesic rats were isolated within a group of 6–7 week STZ-diabetic animals by measuring time of hind paw withdrawal latency, PWL, under thermal stimulation. A diabetic rat was considered to be hypo- or hyperalgesic if its PWL was correspondently higher or lower than the 95 % confidence interval for PWL distribution of the control group, which was 16.6÷19.4 s. Hypoalgesic and hyperalgesic subgroups had correspondently significantly higher (28.3 ± 1.4 s, 32 tests from three rats) or lower (11.8 ± 0.8 s, 19 tests from three rats) averaged PWL compared to control (18 ± 0.7 sec, 28 tests from three rats; *p* < 0.001). Blood glucose level in animals of both diabetic subgroups was significantly higher than in control animals, while mean body weights for both subgroups were not significantly different from control at 6–7 weeks of diabetes (Table 1). These results are similar to ones obtained in the previous work [10], in which downregulation of T-type current was shown in caps^+^ nociceptive neurons. Representative families of total Ba^2+^ currents in the caps^−^lpH^+^ neurons from control and hypoalgesic animals are shown in Fig 3a. It has appeared that the caps^−^lpH^+^ neurons showed upregulation of low voltage-activated Ba^2+^ current in diabetic hypoalgesic animals (Fig 3). The averaged current–voltage curves of a transient component normalized to a cell capacitance are shown in Fig 3b. An increase in TCD in the caps^−^lpH^+^ neurons isolated from hypoalgesic rats was significant and varied from 2.6-fold at a voltage step to −60 mV to 2.8-fold at a voltage step to −40 mV (Fig 3b). Observed upregulation of T-type current in the caps^−^lpH^+^ neurons of 6–7 weeks diabetic hypoalgesic rats was opposite to previously found downregulation of T-type current in the caps^+^ nociceptors of the same animal group [10]. Thus, this upregulation was due to specific sensory modalities of the caps^−^lpH^+^ neurons rather than due to differences in the age of animals or duration of diabetes. It also suggests involvement of TRPV1-independent signal transduction pathway in upregulation of T-type current in the caps^−^lpH^+^ DRG neurons under diabetic conditions. Upregulation of T-type current in the caps^−^lpH^+^ neurons of thermally hypoalgesic diabetic rats implies their involvement in nonthermal nociception.

### Upregulation of T-type current underlies increased excitability of the caps^−^lpH^+^ neurons in diabetic rats

**Fig. 4:**
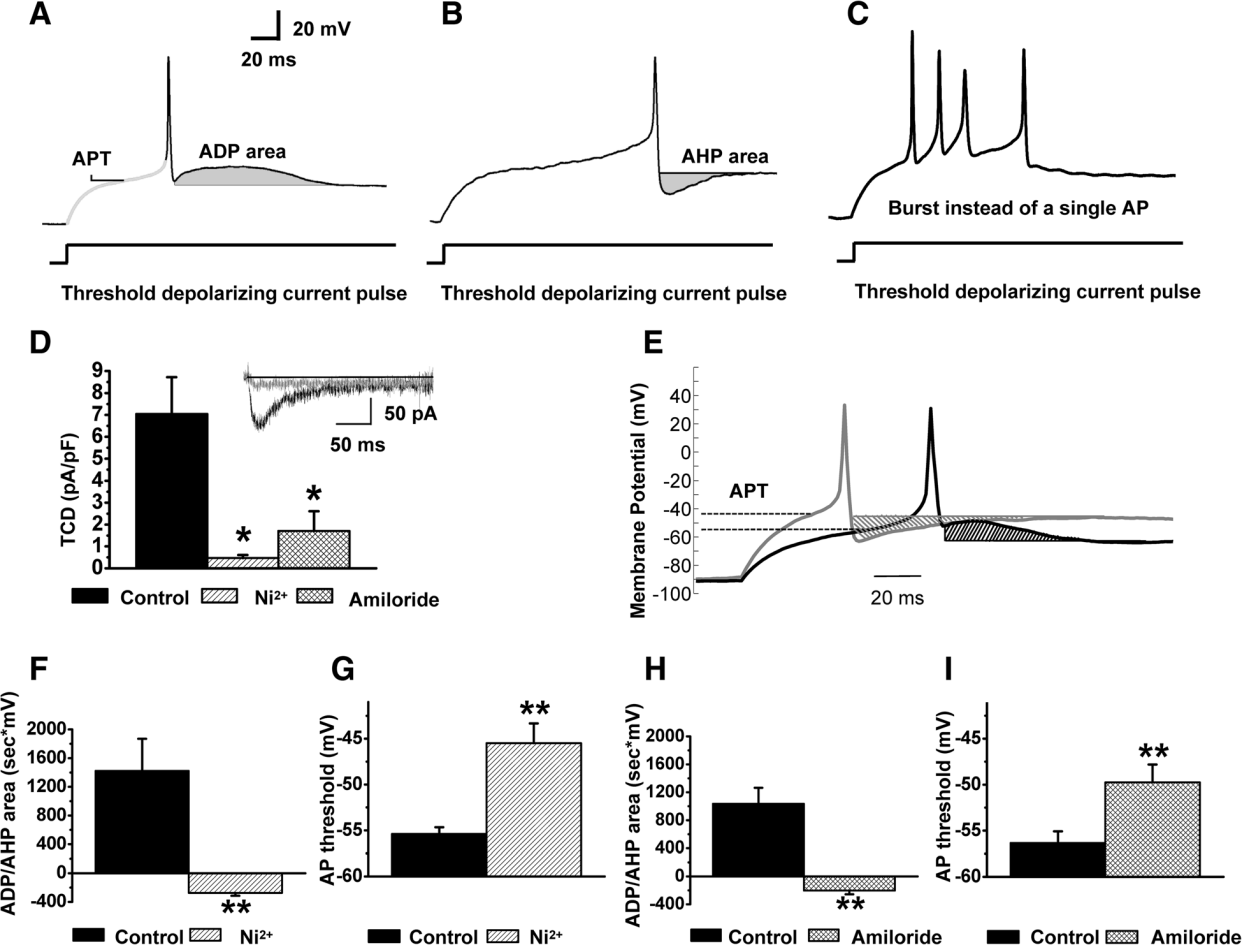
Excitability of the caps^−^lpH^+^ neurons is decreased under T-type channel blockers. T-type channel blockers convert the ADP to AHP and significantly increase the AP threshold for the caps^−^lpH^+^ neurons at concentrations effectively blocking the Ca_v_3.2 isoform of T-type channels. **a,b** Action potential parameters used for estimation of changes in excitability of the caps^−^lpH^+^ DRG neurons (action potential threshold (APT) and after-depolarization/-hyperpolarization areas (ADP/AHP)). **c** An example of an AP burst induced by a threshold stimulation in the caps^−^lpH^+^ neuron of longer-term diabetic rat instead of a single AP observed in the neurons of normal rats. **d** Significant decrease of the Ba^2+^ TCD in the caps^−^lpH^+^ neurons under T-type channel blockers Ni^2+^ (50 μM) and amiloride (1 mM). TCD amplitudes at a voltage step from −100 to −50 mV are presented. Insert: representative traces of Ba^2+^ current before and after application of 50 μM Ni^2+^. **e** A representative example of the AP parameter changes in the caps^−^lpH^+^ neurons of diabetic rats under T-type channel blocker, Ni^2+^ (50 μM). The ADP converted to AHP and the AP threshold increased in 50 μM Ni^2+^ (grey trace) compared to control (black trace). The ADP and AHP areas are cross-hatched. The AP threshold levels are indicated by dashed lines. **f,h** The ADP/AHP area reversed from positive to negative values under 50 μM Ni^2+^ (**f**) and 1 mM amiloride (**h**) reflecting conversion of the ADP to AHP shown on (**e**). **g**,**i** Significant AP threshold increase under 50 μM Ni^2+^ (**g**) and 1 mM amiloride (**i**) Numbers of cells: Ni^2+^ inhibition, *n* = 6 from three rats; amiloride inhibition, *n* = 6 from three rats. *p < 0.05, **p < 0.01

**Fig. 5:**
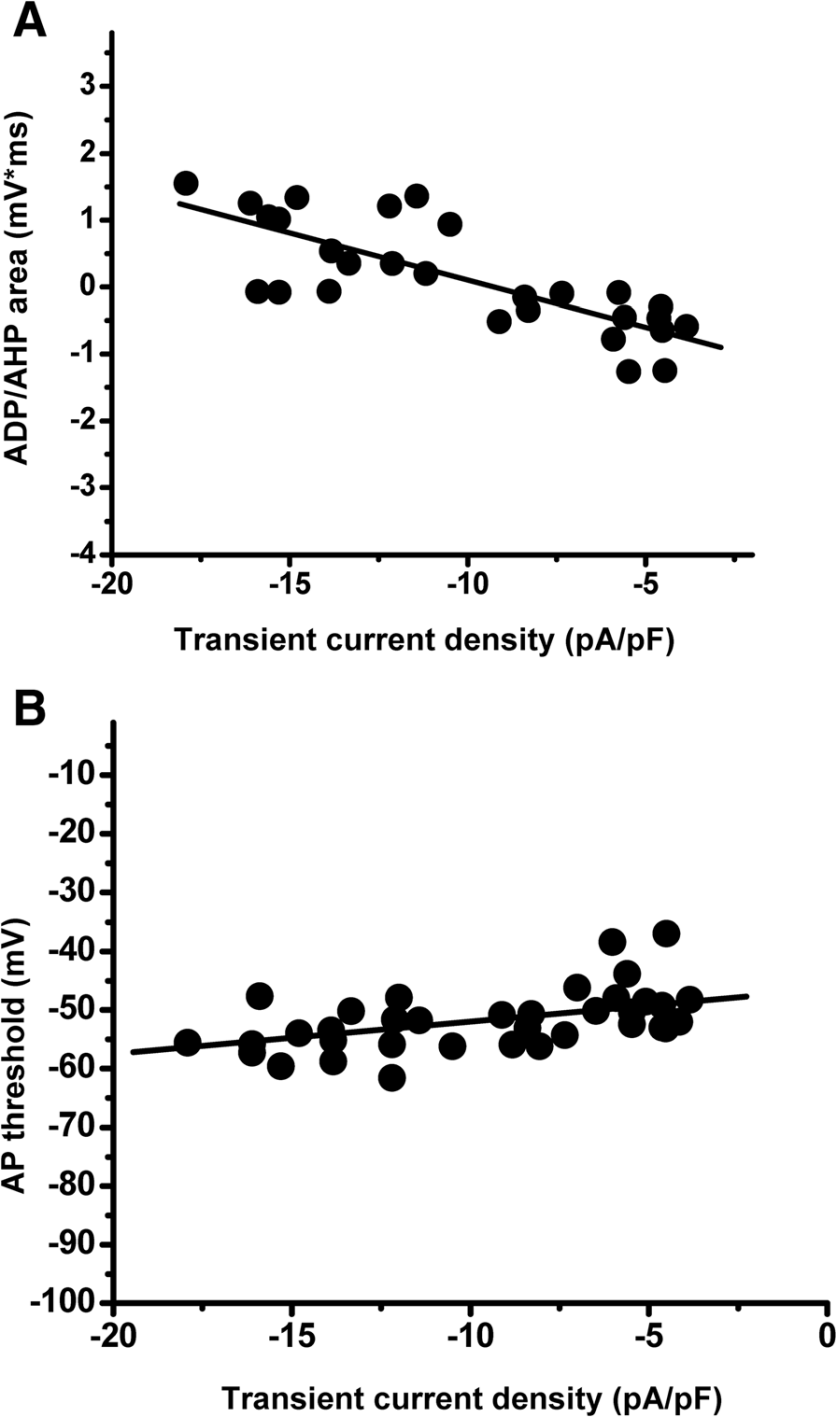
Direct correlation between the AP parameters characterizing neuronal excitability and TCD in the caps^−^lpH^+^ DRG neurons. AP parameters were measured at the AP initiation threshold in Tyrode’s solution. TCD was measured in the same neurons at a voltage step from −100 to −50 mV when the external Tyrode’s solution was changed for TEA-Cl, Ba^2+^-based solution. **a** Significant correlation between the ADP/AHP area and TCD. Pearson coefficient P_c_ = 0.78, *p* < 1.2*10^−5^. Number of cells: *n* = 28 from eight rats **b** Significant correlation between the AP threshold and TCD. Pearson coefficient, P_c_ = 0.52, *p* < 0.0015. Number of cells: *n* = 36 from eight rats

Next, we studied whether upregulation of T-type current observed in the caps^−^lpH^+^ neurons of longer-term diabetic rats resulted in increased excitability of these neurons. Excitability was estimated based on two parameters of neuronal APs: the action potential threshold (APT) determined at a threshold current stimulation evoking an AP (Fig 4a) and the afterhyperpolarization/afterdepolarization (AHP/ADP) area (Fig 4a,b). A fitted part of a trace used to estimate an APT and AHP/ADP area are shown in grey on the Fig 4a,b. An increase in the ADP area as well as a decrease in the APT were considered to be indications of increased neuronal excitability since the former may cause burst firing (Fig 4c, [5]) and the latter should lower sensory input necessary to induce AP.

First, we evaluated variation of excitability parameters due to variation in T-type current in the caps^−^lpH^+^ neurons. Application of T-type channel blocker, Ni^2+^ (50 μM), onto a subset of the caps^−^lpH^+^ neurons having large ADP (and presumably expressing large T-type current [5]) converted the ADP to AHP and increased an AP threshold by 10 mV (Fig 4e-g). The AP threshold trace under Ni^2+^ application (grey trace) is shifted to the left compared to control (black trace) because of the higher value of injected current needed to reach the higher level of AP threshold (Fig 4e). Ni^2+^ application led to a nearly total block of T-type current. Peak transient Ba^2+^ current was inhibited by 93 ± 2 % at a voltage step to −50 mV (Fig 4d), while total Ba^2+^ current was inhibited by 84 ± 2 % at this voltage step. Almost complete block of T-type current by 50 μM of Ni^2+^ suggested that the Ca_v_3.2 T-type channels (IC_50_ = 13 μM [29]) rather than Ca_v_3.1 or Ca_v_3.3 isoforms (IC_50_ = 250 μM and 216 μM, correspondently [29]) mainly contributed to T-type current in the caps^−^lpH^+^ DRG neurons. For a double check of the effect of T-type current block on excitability parameters we applied the other T-type channel blocker, amiloride, which is selective for Ca_v_3.2 T-type channel isoform [30–32] and, to the best of our knowledge, has no significant inhibitory effect on voltage-gated sodium channels at 1 mM concentration. When applied at this concentration, amiloride inhibited Ba^2+^ current by 76 ± 7 % at a voltage step to −50 mV (Fig 4d). Weaker inhibition of T-type current with amiloride resulted in a lesser decrease of the ADP area and lower increase of the AP threshold, demonstrating correlation between the T-type current inhibition and the excitability parameters (Fig 4h, i). So, the highest decrease of the ADP area (by 1704 mV*ms; Fig 4 f) was observed when T-type current was maximally inhibited by Ni^2+^ (by 93 %; Fig 5a) while the ADP area was decreased by 1241 mV*ms; Fig 4h) in case of amiloride application when T-type current was inhibited by 76 % (Fig 4d). Analogous relationship was obtained between the APT and TCD (Fig 4d, g, i). Ni^2+^-induced inhibition of T-type current led to an APT increase by 10 mV (from −55.4 ± 0.7 to −45.5 ± 2.0 mV) (Fig 4g), while amiloride application increased the APT by 7 mV (from −56.3 ± 1.3 to −49.8 ± 2.0 mV) (Fig 4i). A weaker effect of amiloride compared to Ni^2+^ correlated with its weaker inhibition of T-type current (76 ± 7 % for amiloride compared to 93 ± 2 % for Ni^2+^ at a voltage step to −50 mV , Fig 4d).

**Fig. 6:**
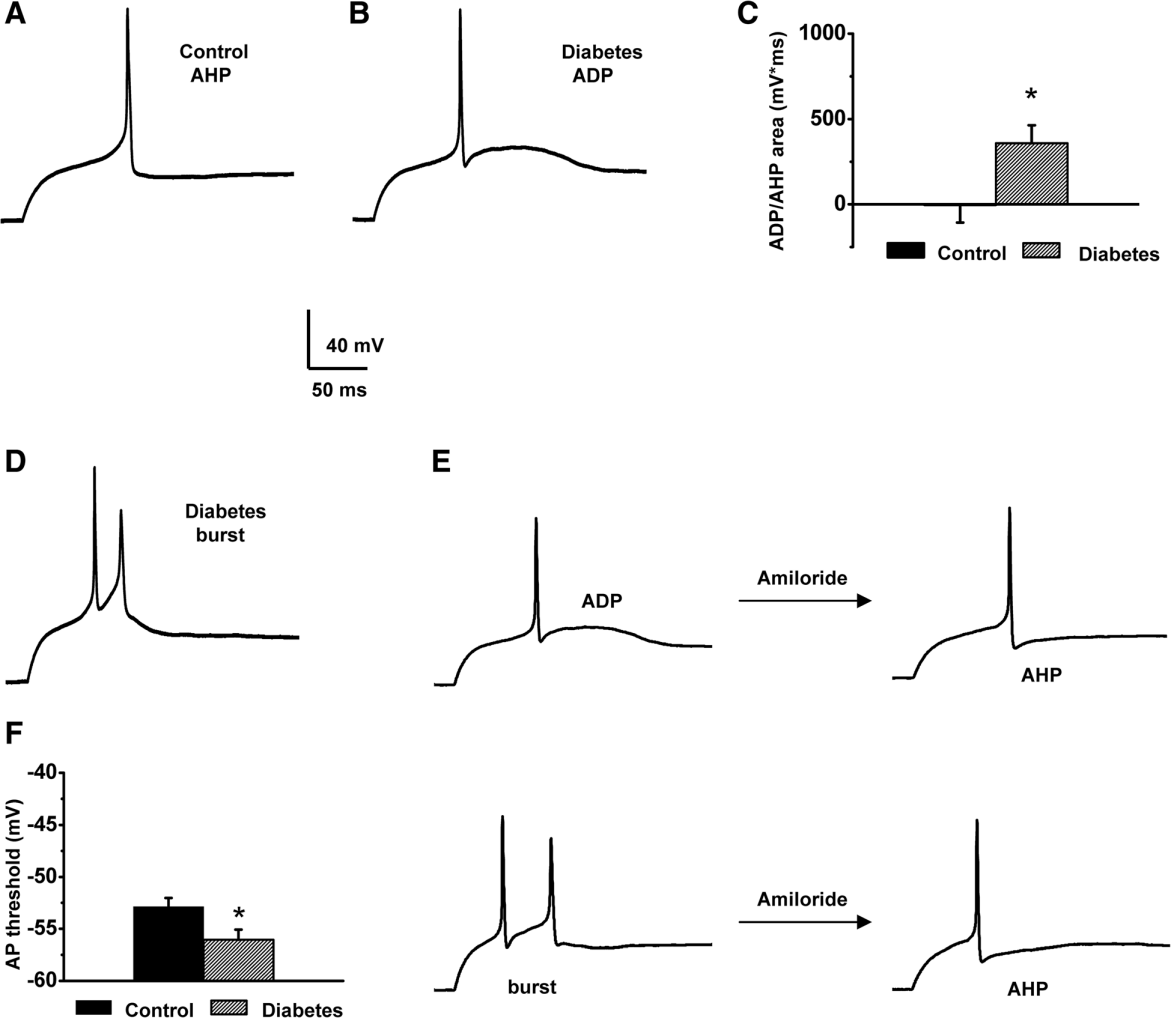
Diabetic-induced changes of the AP parameters for the caps^−^lpH^+^ neurons reflect increased excitability of these neurons in diabetes. **a** A representative AP trace with a slight AHP recorded from the caps^−^lpH^+^ neuron of normal rat. **b** A representative AP trace with a substantial ADP recorded from the caps^−^lpH^+^ neuron of diabetic rat. **c** The ADP/AHP area is significantly increased in diabetes compared to control indicating an increase in excitability of the caps^−^lpH^+^ neurons including probability of their bursting in diabetic conditions. Numbers of cells: control for diabetes *n* = 20 from four rats, diabetes *n* = 28 from four rats, *p* < 0.05. **d** A representative trace of AP bursts generated in 25 % of diabetic neurons (*n* = 28 from four rats) at a threshold current stimulation. At the same time no AP bursts were observed in the caps^−^lpH^+^ neurons of control rats (*n* = 20 from four rats). **e** Application of amiloride converts ADPs (top left) and bursts (bottom left) observed in the diabetic caps^−^lpH^+^ neurons to AHPs (top and bottom right). **f** Diabetes leads to a statistically significant decrease in the AP threshold. Numbers of cells: control for diabetes *n* = 20 from four rats, diabetes *n* = 28 from four rats, *p* < 0.05

Correlation between the ADP/AHP area or the AP threshold and T-type current density was also directly established for the caps^−^lpH^+^ neurons (Fig 5) by measuring AP parameters and TCD for the same neuron in Tyrode’s and TEA-Cl, Ba^2+^-based solutions, respectively. Fig 6 clearly demonstrates a robust correlation between TCD and AP parameters characterizing neuronal excitability. Corresponding Pearson correlation coefficient and level of significance for correlation of the ADP/AHP area and the AP threshold with the TCD were 0.78, *p* < 1*E^−5^ and 0.7, *p* < 0.002, respectively. Thus, T-type current in the caps^−^lpH^+^ neurons is significantly correlated with the neuronal excitability parameters.

This correlation between the values of T-type current and excitability parameters assumes increased excitability of the caps^−^lpH^+^ neurons in diabetes when upregulation of T-type current is observed (Fig 2b). Fig 6a,b show representative AP traces generated in control and diabetic neurons at a threshold current stimulation from −90 mV. More than half of diabetic caps^−^lpH^+^ neurons (57 %, *n* = 28, four rats) revealed a prominent ADP (Fig 6b) whereas majority of control cells (75 %, *n* = 20, four rats) displayed an AHP (Fig 6a). Statistically it was manifested in a significant increase of the ADP/AHP area (by 467 mV*ms) in diabetes compared to control (Fig 6c). An increased ADP/AHP area resulted in a burst generating capability of the diabetic caps^−^lpH^+^ neurons. These neurons showed a burst of two spikes with a second spike crowning an ADP hump (7 out of 28 tested neurons; Fig 6d), while no bursts were recorded in the caps^−^lpH^+^ neurons of control rats (*n* = 20, four rats). Application of T-type channel blocker amiloride eliminated the hump at a threshold current stimulation (Fig 6e), and abrogated the second spike generated at a slightly higher depolarization (Fig 6e) implying that T-type channel upregulation results in higher excitability of the caps^−^lpH^+^ neurons under longer-term diabetes. Another excitability parameter, the AP threshold, was also significantly decreased (from −53 ± 0.8 in control to −56 ± 1 mV in longer-term diabetes; Fig 6 f) suggesting that weaker sensory stimuli would be enough to evoke AP in the caps^−^lpH^+^ neurons of diabetic rats.

Thus, the obtained results demonstrate increased excitability of the caps^−^lpH^+^ neurons in the longer-term diabetic rats caused by increased functional expression of the Ca_v_3.2 T-type channels in these neurons.

### Sodium voltage-operated currents do not contribute to increased excitability of the caps^−^lpH^+^ DRG neurons in longer-term diabetes

**Fig. 7:**
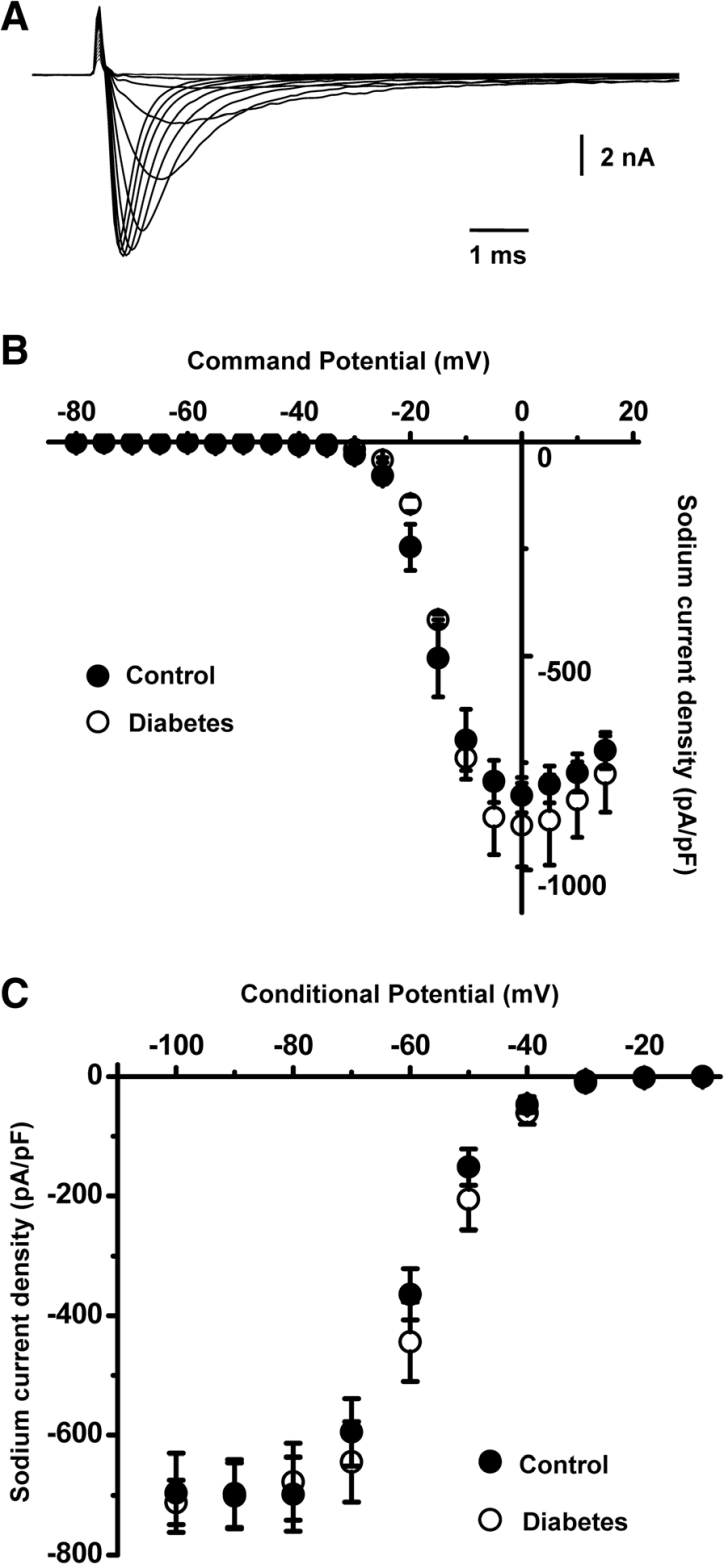
Total voltage-gated Na^+^ current is not significantly changed in the caps^−^lpH^+^ neurons of diabetic rats. **a** A representative example of Na^+^ currents obtained using a voltage-dependent activation protocol. **b** Steady-state activation curves of Na^+^ current in the caps^−^lpH^+^ neurons of diabetic and control rats. **c** Steady-state inactivation curves of Na^+^ current in the caps^−^lpH^+^ neurons of diabetic and control rats. *n* = 7 from three rats for diabetic and control groups in B and C. Differences in peak current densities were insignificant (*p* > 0.05). These results clearly demonstrate a lack of Na^+^ current modulation under diabetic conditions

Diabetes-induced remodeling of sodium channels may also potentially increase the neuronal excitability [9] and account for the increased excitability of the caps^−^lpH^+^ neurons in the longer-term diabetic rats. To find out whether sodium current might contribute to the increased excitability of the caps^−^lpH^+^ neurons in longer-term STZ diabetes this current was compared in the neurons of control and diabetic rats. No significant diabetes-induced changes in sodium current density were found in a whole range of tested membrane potentials in protocols for channel activation and inactivation (Fig 7). In fact, the current density in the diabetic neurons was slightly and insignificantly lower compared to control in a range of low voltage membrane potentials (up to −40 mV), at which T-type current contributes to the neuronal excitability (Fig 7b). No significant changes in parameters of Boltzmann fitting functions, *G*_*max*_, *I*_*max*_, *k* and *V*_*50*_, were found in diabetes versus control for steady-state activation and inactivation of sodium current (data not shown).

**Fig. 8:**
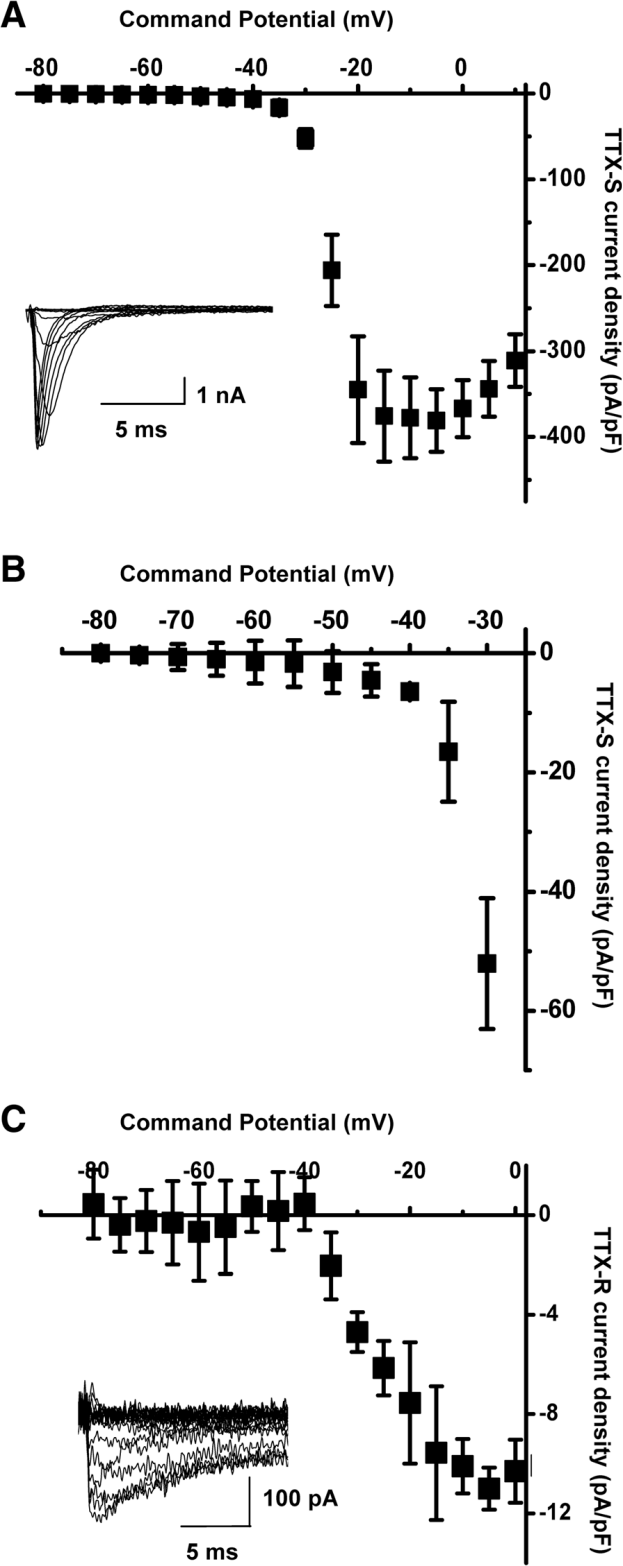
TTX-sensitive and TTX-resistant Na^+^ currents do not contribute to neuronal excitability in the caps^−^lpH^+^ neurons. **a** Steady-state activation of TTX-sensitive, TTX-S, component of Na^+^ current. Insert: representative traces of TTX-S Na^+^ current **b** A part of a low voltage region from A is presented in a larger scale demonstrating negligible values of TTX-S current at depolarization steps in a range of −70 - -50 mV (*n* = 9 from three rats). **c** Steady-state activation curve of TTX-resistant, TTX-R, component of Na^+^ current demonstrating its activation threshold at −35 mV (*n* = 9 from three rats). Insert: representative traces of TTX-R Na^+^ current. Altogether these results demonstrate a lack of low-threshold TTX-R current and substantially lower density of TTX-S current compared to T-type current [39] at depolarization steps up to -40 mV

TTX-resistant sodium channels (TTX-R) along with TTX-sensitive channels (TTX-S) are widely expressed in small DRG neurons and are implicated in the molecular mechanisms of nociception and pain [33–35]. Particularly, TTX-R sodium channels encoded by Na_v_1.9 play a similar role to that of T-type channels in lowering the AP threshold and promoting burst discharges [36–38]. Therefore, we pharmacologically isolated TTX-S and TTX-R components of sodium current in the caps^−^lpH^+^ neurons (Fig 8) in order to check whether both components are present in these neurons and whether they may substantially contribute to neuronal excitability. To isolate the TTX-R component a subtraction procedure was applied to current traces recorded in a series of different external solutions (see Materials and Methods). TTX-R current appeared to be small even at the maximum of *I-V* curve (<12 pA/pF) and had an activation threshold around −35 mV (Fig 8c), lacking a low voltage-activated component conducted by Nav1.9 channels and being represented solely by high voltage-activated current conducted by Nav1.8 channels. Thus, it could not affect either the ADP area or the AP threshold in the caps^−^lpH^+^ neurons in the −80 - -40 mV range of depolarization, where the sodium current is represented by its TTX-S component. This component had a shallow slope of activation curve (from 0 to about 1.6 pA/pF) in a range of test potentials from −80 to −50 mV (Fig 8a, b) and a density substantially less than one of T-type Ca^2+^ current. T-type current density estimated at the level of an AP threshold of −53 mV in Tyrode’s solution was ~6 pA/pF (Fig 2b; assuming I_Ba_:I_Ca_ = 1.2 [39])) and, therefore, TTX-S could not determine the AP threshold. Thus, we have found that voltage-operated sodium channels could not substantially influence the values of the ADP area and the APT in the caps^−^lpH^+^ neurons and were not upregulated in the longer-term diabetic rats. At the same time T-type Ca^2+^ channels were upregulated and definitely contributed to increased excitability of the caps^−^lpH^+^ neurons in longer-term diabetes.

## Discussion

The main finding of this work is that nociceptive caps^−^lpH^+^ DRG neurons exhibit increased excitability in longer-term diabetes due to upregulation of T-type channels. To the best of our knowledge, this is the first study, in which upregulation of T-type calcium channels has been shown under conditions of thermal hypoalgesia in a specific type of nociceptors. This finding supports an idea that remodeling of T-type channels in these functionally specific nociceptive neurons may underlie painful states in longer-term diabetes.

### Specific type of DRG neurons reveals upregulation of T-type current in longer-term diabetic rats and in thermally hypoalgesic diabetic rats

In this study we have isolated a particular type of small-sized caps^−^lpH^+^ DRG neurons. These neurons belong to a population of very small DRG neurons based on size/capacitance classification used by Petrusca et al. [21]. Small size and specific action potential parameters of these neurons, including long AP width, high AP overshoot and slow AHP recovery, allow us to classify them as C-fiber nociceptors [25]. Additionally, these neurons have specific traits of NGF-dependent peptidergic nociceptors such as IB4-negative capsaicin-insensitive phenotype, expression of TTX-resistant high-threshold sodium (Na_v_1.8) and ASIC channels and absence of low-threshold sodium channels (Na_v_1.9) [26, 27, 40–42]. Thus, the caps^−^lpH^+^ neurons can be classified as NGF-dependent peptidergic ASIC-expressing C-fiber nociceptors.

Our results demonstrate that upregulation of T-type current under diabetes is not accompanied by significant changes in its steady-state or kinetic parameters. It implies an increase in either density of functionally active channels or their single channel conductance without changes in their gating properties. Classifying the caps^−^lpH^+^ neurons helps to better understand possible pathways of T-type current upregulation in these neurons that leads to their increased excitability in diabetes. Assumed NGF dependence of the caps^−^lpH^+^ DRG neurons implies possible involvement of NGF-dependent signal transduction pathways in activation of T-type channels expression and remodeling as they were found to be involved in generation of inflammatory pain [43–45] and are activated at a painful stage of diabetes [46]. The other reason for T-type channel upregulation may be related to acidosis and activation of ASIC-dependent signal transduction pathways at an advanced stage of diabetes [14, 47]. Thus, the caps^−^lpH^+^ neurons can potentially be involved in nociception by means of T-type current upregulation mediated by ASIC- and NGF-dependent pathways. Additionally, accessory HVA Ca^2+^ channel subunit α2δ was found to increase T-type current density [48], so elevated expression of the Ca_v_3.2 channels could be induced by upregulation of the α2δ subunit expression found in STZ-induced diabetes [49].

Differential modulation of T-type channels in caps^+^ [10] and caps^−^lpH^+^ (this study) neurons in the same thermally hypoalgesic diabetic rats implies that T-type current expression in diabetes depends on neuronal sensory modality rather than on pathogenic conditions (hyperglycemia, ischemia, etc.) common for all types of DRG neurons. That is, diabetes-induced modulation of T-type channels may depend upon TRPV1 and ASIC sensory pathways in caps^+^ and caps^−^lpH^+^ neurons, respectively.

Previous studies have shown that thermal hyperalgesia/hypoalgesia in diabetic animals correlates with T-type channels upregulation/downregulation in caps^+^ DRG neurons [10]. Multiple behavior tests during STZ-induced diabetes development has proven that thermal hyperalgesia developing at an early stage of diabetes (2–4 weeks) [4, 5] converts to hypoalgesia by 8–12 weeks of diabetes [6, 7] (for review see [8]) while other pain symptoms (spontaneous pain, mechanical hyperalgesia, and tactile allodynia) persist through this later stage of diabetes development [7, 8, 12]. Upregulation of T-type current found in the caps^−^lpH^+^ neurons of long-term diabetic (9–13 weeks) and thermally hypoalgesic diabetic rats strongly suggests their involvement in nonthermal nociception persisting at a later stage of diabetes.

### Upregulation of Ca_v_3.2 T-type channels leads to increased excitability of the caps^−^lpH^+^ DRG neurons and may cause pain in longer-term diabetes

Previously it has been found, that Ca_v_3.2 isoform of T-type channels is the major contributor to T-type current, which is upregulated in small- and medium-sized DRG neurons of diabetic rats. It has been shown that this upregulation is necessary for development of hyperalgesia [4, 5]. In this work, almost complete inhibition of T-type current by 50 μM of Ni^2+^ (Fig 4d) suggests that the Ca_v_3.2 isoform (IC_50_ = 13 μM [29]) makes a major contribution to T-type current in the caps^−^lpH^+^ neurons since other two T-type channel α_1_ subunit isoforms, Ca_v_3.1 and Ca_v_3.3, are less susceptible to inhibition by Ni^2+^ (IC_50_ = 250 μM and 216 μM, correspondently [29]). Efficient conversion of ADP to AHP and decrease of AP threshold in the caps^−^lpH^+^ neurons of diabetic rats under 50 μM of Ni^2+^ (Fig 4e) and selective Ca_v_3.2 blocker, amiloride [30–32] also proves that Ca_v_3.2 channels determine excitability of the neurons under STZ-induced diabetes.

The major modulatory role of T-type channels in AP generation threshold compared to TTX-R and TTX-S sodium channels in the caps^−^lpH^+^ neurons is determined by a substantially higher value of T-type current at depolarization steps equal or lower than the AP threshold level. For example, the T-type current density is about 4 times higher than the density of sodium current at membrane depolarization to potentials close to AP generation threshold for the caps^−^lpH^+^ neurons (see Results, Fig 2b and Fig 8a, b). It is in contrast to other types of DRG neurons where T-type currents are small and sodium channels play a major modulatory role in defining an AP threshold. For example, TTX-R sodium channels determine an AP threshold in Type 2 DRG neurons [50].

Upregulation of T-type Ca^2+^ current in the caps^−^lpH^+^ neurons of diabetic rats shown in this study could potentially modulate peripheral nociceptive input to the dorsal horn at the level of peripheral and central terminals of these neurons. It has been shown that pain stimuli are detected by transducer channels (TRP class thermal/chemical receptors, ASICs, P2X3, etc.) and converted to subthreshold depolarization [51]. This depolarization reaches an AP threshold by means of generator potentials, the main role in which in nociceptive DRG neurons is supposed to be played by some voltage-gated sodium channels [51]. They are expressed in nerve endings of C-fibers *in vivo* [52] and are present *in vitro* in distal axons of small DRG neurons at densities allowing amplification of a subthreshold depolarization [53]. In this study we have found that somatic subthreshold depolarization may reach the AP threshold in the caps^−^lpH^+^ neurons due to T-type calcium rather than sodium channels (Fig 4e, g, i). Moreover, T-type channel upregulation in longer-term diabetes would naturally cause peripheral sensitization of the caps^−^lpH^+^ DRG neurons by producing AP bursts instead of single APs (Fig 6d). Here we have demonstrated that diabetes-induced upregulation of T-type channels in the caps^−^lpH^+^ neurons leads to an increase in percentage of neurons demonstrating double-spike bursts from 0 % in control to 25 % in longer-term diabetes. This may certainly contribute to painful sensations experienced by diabetic patients since AP bursts are most effective in producing dorsal horn neuronal plasticity [54] and supporting chronic pain states [55, 56]. It is important to note that expression of the Ca_v_3.2 isoform of T-type Ca^2+^ channels in peripheral axons of DRG neurons have been recently reported [57] additionally confirming that these channels may amplify generator potentials in peripheral endings of the caps^−^lpH^+^ DRG neurons.

Diabetes-induced changes in functional expression of T-type channels in the caps^−^lpH^+^ neurons might also contribute to pain processing at the level of central terminals of these neurons. It has been shown that T-type channels regulate the frequency rather than the amplitude of miniature excitatory postsynaptic currents recorded in dorsal horn neurons from acute spinal cord slices [58]. The effect was specific for superficial dorsal horn neurons compared to deeper non-nociceptive lamina [58]. These findings provide evidence for the role of presynaptic T-type channels in synaptic transmission between nociceptive DRG and secondary dorsal horn neurons. Thus, diabetes-induced T-type channel remodeling observed in this work may modify synaptic transmission between the cap^−^lpH^+^ and dorsal horn neurons at the level of central terminals in this way contributing to an increased level of pain in longer-term diabetes.

## Conclusions

Caps^−^lpH^+^ DRG neurons exhibit increased excitability in long-term diabetic and thermally hypoalgesic diabetic rats and likely contribute to nonthermal nociception at the later-stage diabetes. Their hyperexcitability is caused by upregulation of T-type calcium channels mainly represented by Ca_v_3.2 subtype. Thus, Ca_v_3.2 channels of caps^−^lpH^+^ nociceptive DRG neurons can be a therapeutic target for treating pain symptoms in later-stage diabetic patients.

## Methods

All animal care and handling was done in accordance with protocols of the Animal Care and Use Committee at Bogomoletz Institute of Physiology.

### Induction of experimental diabetes

Diabetes was induced by injection of STZ in young (3 weeks old to obtain animals with 6–7 weeks long diabetes) or adult (5–6 months old to obtain animals with 9–13 weeks long diabetes) male Wistar rats (50 mg/kg, i.p.). STZ was prepared in 100 mM citric acid, pH 4.2, on ice. Blood glucose level was measured on third day with glucometer Glucotrend (Boehringer Mannheim, Germany). If a blood glucose level was below 25 mM, an additional STZ injection was given (25–40 mg/kg, i.p.) that increased probability of hyperglycemia development in those animals. The blood glucose level was measured again on third day, during the course of disease development and on a day of DRG isolation. All animals used in our experiments had the blood glucose level higher than 25 mM after establishment of hyperglycemia and before DRG isolation. Animals in the control group were of the same age as ones in the diabetic group and were injected with a saline instead of STZ.

### Assessment of thermal sensitivity

Nociceptive responses to thermal stimuli were measured at room temperature using a paw thermal stimulation system (Uga Basile, Italy) consisting of a plastic chamber with a transparent glass floor and radiant heat source positioned beneath the floor to deliver a thermal stimulus to the plantar side of the hind paw. Paw withdrawal latency (PWL) was assessed in seconds by the automatic timer as a time interval between a start of a thermal stimulus delivery and a paw withdrawal, when a photocell detected interruption of a light beam reflection. PWL was measured in control and diabetic rats a day before the experiment. One-way ANOVA was used to reject the null hypothesis that control and diabetic groups have the same mean PWL. Diabetic rats were considered hypoalgesic if their PWL was higher than the right bound of the 95 % confidence interval for PWL distribution obtained for the control animals (see [10] for details). Similarly, the diabetic rats were considered hyperalgesic if their PWL was lower than the left bound of the 95 % confidence interval obtained for the control rats.

### Acutely dissociated DRG neurons

Lumbar L_4_-L_6_ DRG were isolated from diabetic or control animals of the same age. Ganglia were treated for 30 min at 36 ° C with 0.15 % of the type 4 collagenase, 250 U/mg (Worthington, USA) and 0.15 % trypsin, 204 U/mg (Worthington, USA) in the Tyrode’s solution containing in mM 140 NaCl, 4 KCl, 2 MgCl_2_, 2 CaCl_2_, 10 glucose and 10 HEPES, adjusted to pH 7.4 with NaOH. They were washed from enzymes in fresh Tyrode’s solution and mechanically dissociated by triturating them through a series of Pasteur pipettes polished to several narrowing diameters. Isolated cells were collected by centrifugation, resuspended in fresh Tyrode’s solution and kept at room temperature until use in electrophysiological experiments during 3–8 h after isolation.

### Electrophysiology

Whole-cell electrophysiological recordings were performed at room temperature using RK-400 patch-clamp amplifier (Intracel, UK) conventional techniques. Voltage and current commands and digitization of membrane voltages and currents were controlled using an ITC-16 interfaced with TIDA data acquisition software (HEKA, Germany) running on a PC computer. Data analysis was accomplished using MATLAB 7.5 (MathWorks, USA) and Origin 8.0 (Microcal Software, USA). Currents were low-pass filtered at 2–5 kHz. Series resistance (Rs) and capacitance (Cm) values were taken directly from dials of the amplifier after electronic subtraction of capacitive transients. Series resistance was compensated to the maximal extent possible (80-90 %). Linear leak subtraction was used for all voltage-clamp recordings.

Patch pipettes were pulled from borosilicate glass microcapillary tubes (Sutter Instrument, USA) using P-97 Flaming/Brown micropipette puller (Sutter Instrument, USA). They had resistance 2 to 3 MΩ when filled with internal solutions. Two internal solutions were used in the experiments. Solution 1 was used for typing neurons, recording Ba^2+^ currents and in all current-clamp experiments. It contained the following (in mM): 120 KCl, 4 MgATP, 30 HEPES, 2.25 CaCl_2_, 10 EGTA, 10 NaCl, pH 7.4 with KOH, 296 mOsm. Solution 2 was used to record Na^+^ currents and contained the following (in mM): 120 CsCl, 20 HEPES, 10 EGTA, 5 Mg-ATP, 0.4 Na-GTP, pH 7.2 with CsOH. DRG neurons were clamped in a whole-cell configuration and typed in Tyrode’s solution described above. All current-clamp experiments were done after typing using the same external solution with an addition of T-type channel blockers, if necessary. To record Ba^2+^ currents the Tyrode’s solution in a bath was changed for the following (in mM): 165 tetraethylammonium (TEA)-Cl, 10 HEPES, 2 BaCl_2_, pH 7.4 with TEA-OH, 305–315 mOsm. In order to isolate tetrodotoxin-sensitive (TTX-S) and resistant (TTX-R) components of Na^+^ currents recordings were done sequentially in (i) Tyrode’s solution, (ii) Tyrode’s solution plus 1 μM TTX, and (iii) finally in a solution containing (in mM): 150 choline chloride, 10 HEPES, 2 CaCl_2_, 2 MgCl_2_, 4 KCl, pH 7.4 with Tris-base. TTX-S current traces were obtained by subtracting recordings performed in the second solution from the ones performed in the first. TTX-R current traces were obtained by subtracting recordings performed in the third solution from the ones performed in the second. To compare sodium currents in neurons of diabetic and normal rats recordings were done in Tyrode’s solution with the addition of La^3+^ (10 μM) to block calcium channels and TEA (20 mM) to block potassium channels. The internal solution 1 (KCl-based) had a predicted junctional potential of about −5 mV against the Tyrode’s solution and about −9 mV against the TEA-based external solution. The internal solution 2 (CsCl-based) had a predicted junctional potential of about −8 mV against the Tyrode’s solution. Clempex 8.2 software was used to calculate predicted junctional potentials. Reported membrane potentials were not corrected for these values.

Neurons were typed using voltage-step stimulation protocols 1 and 2 taken from [21]. Protocol 2 was used as a routine to differentiate types of neurons. It included depolarization steps from a holding potential of −100 mV (3.5 s) to test potentials from −60 to +40 mV (200 ms) with 20 mV increments. Protocol 1 was used to examine hyperpolarization activated currents included hyperpolarization steps from a holding potential of −60 mV (4 s) to test potentials from −60 to −110 mV (500 ms) with 10 mV increments. Protocol for activation of Ba^2+^ currents included depolarization steps from a holding potential of −100 mV (3.5 s) to test potentials from −80 to 0 mV (250 ms) with 10 mV increments. Protocol for activation of sodium currents was similar to the later one, but test potentials ranged from −80 to +15 mV with 5 mV increments. The steady-state inactivation protocol for Ba^2+^ currents included depolarization steps to a test potential of −40 mV (250 ms) from a holding potential ranging from −100 to −40 mV (3.5 s) with 10 mV increments. The steady-state inactivation protocol for Na^+^ currents included depolarization steps to a test potential of −10 mV from a holding potential ranging from −100 to −10 mV (3.5 s) with 10 mV increments.

The reverse potential for a transient component of Ba^2+^ current, mainly mediated by the Ni^2+^-sensitive Ca_v_3.2 channels, was obtained using KCl-based internal solution and Ba^2+^-,TEA-Cl-based external solution by extrapolation of Ni^2+^-sensitive current–voltage curve to a zero current value. Ni^2+^-sensitive current was obtained by subtraction of recordings performed in external solutions with and without Ni^2+^ (50 μM).

The protocol for action potentials (APs) generation consisted of a series of increasing current injections (350 ms) evoking incremental depolarization (2–3 mV per each step) from a holding potential of −90 mV.

### Analysis

Two parameters of AP were used in order to compare excitability of DRG neurons in normal and diabetic conditions. The first parameter, the AP threshold, was defined as a potential at an inflection point in a trace part preceding an AP upstroke at a threshold current stimulation. This part of a trace was fitted by the sum of two exponentials: one - to fit a time course of subthreshold membrane depolarization [59] and another - to fit a part of a trace in the interval between an AP threshold and an AP upstroke [60, 61]. An inflection point was determined as the one, at which a second derivative of a fitting function turned zero. A second AP parameter determining neuronal excitability used in this study was an area under the afterdepolarization potential (ADP) or above the afterhyperpolarization potential (AHP) determined at a threshold depolarization invoking an AP. The ADP/AHP area was measured in mV*ms.

Total Ba^2+^ current was separated into transient and persistent components (Fig3a). A transient component was calculated as a difference between total current and mean current at the end of a voltage step. The current at the end of a voltage step was considered as a persistent component. Voltage-activated conductance of transient current, *G(V),* was calculated as peak density of transient current divided by a driving force *V – V*_*r*_, where *V* is a membrane potential and *V*_*r*_ is a reversal potential of transient current. *V*_*r*_ was found by extrapolation of the current–voltage curve to a zero current value, as described above in section Electrophysiology*,* to be equal +30 mV. *G(V)* and steady-state inactivation *I(V)* were described with single Boltzmann distributions of the following forms:

1 $$ G(V) = {G}_{max}/\left(1 + exp\Big(-\left(V-{V}_{50}\right)/k\right) $$

and

2 $$ I(V) = {I}_{max}/\Big(1 + exp\left(\left(V-{V}_{50}\right)/k\right) $$

In these equations *G*_*max*_ and *I*_*max*_ are maximal conductance and steady-state inactivation current density, correspondently; *V*_*50*_ is a membrane potential at which a half of the *G*_*max*_ or *I*_*max*_ values are reached and *k* is a slope factor. In a range of voltage steps from −80 to −40 mV both transient and persistent components of total Ba^2+^ current were mediated mainly by the T-type channels (data not shown). Beginning with a voltage step to −30 mV N- and other types of high voltage-activated (HVA) currents were found to substantially contribute to the total Ba^2+^ current (data not shown) so that a transient component mediated by the T-type channels could not be determined at this voltage step without blocking HVA channels. We estimated a transient component value at a voltage step to −30 mV from the best Boltzmann function fit of a steady-state transient current activation curve (*G(V)/Gmax*) in a range of −80 to −30 mV. Fitting was done with a MATLAB *fit function* (MathWorks, USA) using a nonlinear least-squares method and the best fit was determined by the highest score of the goodness of fit parameter *adjrsquare*. At the end of fitting procedure the parameter *adjrsquare* was in a range of 0.980 - 0.993.

Statistical comparisons were done using a paired Student’s t-test when T-type current inhibitors were used and unpaired Student’s t-test when comparing data obtained from diabetic and normal rats. One-Way ANOVA was used to find out that measured values were not significantly different between animals within control and diabetic groups and could be combined into cumulative control and diabetic samples to be compared with the Student’s t-test. Intergroup differences with *p* < 0.05 were considered to be significant. Factors that might influence the difference between control and “diabetic” neurons such as sex, litter or age were excluded by the following design. Male siblings from several litters with approximately the same age (the difference was within 1 week) were seperated into control and “diabetic” groups that were blindly taken care of side by side. Direct correlation between an ADP/AHP area or an AP threshold and T-type current values was established using Pearson correlation test that was done on data pooled together from diabetic and control animals.

## Abbreviations

STZ: Streptozotocin (N-[methylnitrosocarbamoil]-D-glucosamine)
PWL: Paw withdrawal latency
Caps^+^: Capsaicin-sensitive
Caps^−^: Capsaicin-insensitive
lpH^+^: Low-pH-sensitive
TCD: Transient current density
APb: Action potential duration at the base
AHP80: Afterhyperpolarization at 80 % recovery
TTP: Time to peak
APT: Action potential threshold
